# Research on the local path planning of an orchard mowing robot based on an elliptic repulsion scope boundary constraint potential field method

**DOI:** 10.3389/fpls.2023.1184352

**Published:** 2023-07-21

**Authors:** Wenyu Zhang, Ye Zeng, Sifan Wang, Tao Wang, Haomin Li, Ke Fei, Xinrui Qiu, Runpeng Jiang, Jun Li

**Affiliations:** ^1^ College of Engineering, South China Agricultural University, Guangzhou, China; ^2^ Guangdong Laboratory for Lingnan Modern Agriculture, Guangzhou, China

**Keywords:** mowing robot, artificial potential field, path planning, local minimum, boundary potential field

## Abstract

In orchard scenes, the complex terrain environment will affect the operational safety of mowing robots. For this reason, this paper proposes an improved local path planning algorithm for an artificial potential field, which introduces the scope of an elliptic repulsion potential field as the boundary potential field. The potential field function adopts an improved variable polynomial and adds a distance factor, which effectively solves the problems of unreachable targets and local minima. In addition, the scope of the repulsion potential field is changed to an ellipse, and a fruit tree boundary potential field is added, which effectively reduces the environmental potential field complexity, enables the robot to avoid obstacles in advance without crossing the fruit tree boundary, and improves the safety of the robot when working independently. The path length planned by the improved algorithm is 6.78% shorter than that of the traditional artificial potential method, The experimental results show that the path planned using the improved algorithm is shorter, smoother and has good obstacle avoidance ability.

## 1 Introduction

With the development of science and technology, mobile robots are increasingly used in agriculture. In orchards, mowing robots with autonomous navigation ability are a hot research topic. As a key autonomous navigation technology, path planning has attracted increasing attention from researchers.

According to the degree of a mobile robot’s mastery of the information in an area, path planning can be divided into two types: one is global path planning based on complete area information (Li et al., 2022), and the other is local path planning based on local area information (Wu et al., 2022). Algorithms to solve global path planning include particle swarm optimization (PSO) (Delice et al., 2017; Wang et al., 2018), visibility methods (Zimmermann and König, 2016; Salman et al., 2023), and link graph methods (McCammon and Hollinger, 2021) and topology method (Jin and Choi, 2011). Algorithms to solve local path planning include artificial potential field methods (Khatib, 1986), the ant colony algorithm (Gao et al., 2023; Li et al., 2023), the A* algorithm (Zhang et al., 2022), artificial immune methods (Lin et al., 2023) and rolling window methods (Xin et al., 2023). Real-time mowing robot obstacle avoidance mainly utilizes local robot path planning algorithms. Because of the advantages of a simple structure, easy understanding, small calculation and real-time capability, artificial potential field methods are widely used in the robot field.

The basic idea of an artificial potential field (APF) method is constructing a virtual APF that senses the positions of the robot, obstacles and target points in an environment using sensors so that the mobile robot can be influenced by the target points and obstacles at the same time. In the potential field, the robot is attracted by the target points and moves toward them while being repelled by the obstacles and moves away from them. Therefore, under the action of this resultant force, the robot avoids obstacles and moves toward the target points, thus planning a collision-free path. Compared with other classical obstacle avoidance algorithms, an APF method has the advantages of fewer calculations, solving local obstacle avoidance problems and solving sudden challenges. Therefore, this algorithm is widely used in obstacle avoidance methods. However, an APF method has the following obvious disadvantages:

1. Target unreachable problem: When the robot is far away from a target point, the attraction will become extremely large. If the relatively small repulsion force can be ignored, the robot may encounter obstacles on its path. When there are obstacles near the target point, the repulsion force will be very large, and the attraction will be relatively small, making it difficult for the robot to reach the target point. When the distance between the robot and the target point is very close, if there is an obstacle near the target point, the attraction on the robot is approximately zero relative to the large repulsion, and the robot will always wander around the target point and cannot reach the target point.
2. Local minimum problem: The robot relies on the overlapping of the potential fields detected from all directions to obtain the overlapping field, and the direction and size of the overlapping field are used to determine the next trajectory. However, if the overlapping field is close to zero, the robot will not move and stop.
3. Poor adaptability in a complex environment: The more obstacles there are in the overlapping field, the higher the probability that the overlapping field is zero, and the easier it is to stagnate, leading to the local minimum problem.

In this regard, many scholars have invested much energy in research and improvement. Based on an artificial immune algorithm, Hou YB (Hou et al., 2012) adopted a potential field function method in an APF method to easily obtain the optimal path and improve the quality of path planning. Q. Song (Q. Song et al., 2012) To effectively solve the local minimum problem of APF methods, the force function of the potential field was improved using a velocity vector, and the repulsive potential field coefficient was adjusted in real time by combining it with a fuzzy control algorithm, which overcomes the robot easily falling into a local minimum and alleviates the oscillation problem. Li G (Li et al., 2013) proposed an improved APF method based on a regression search method, redefined the potential field function to solve the local minimum and oscillation problems, improved a wall-following method to solve the unreachable problem, and optimized the planned path using a regression search algorithm to obtain a better and shorter effective path. To solve the problems of local minimum and inefficiency of classical APF methods, Abdalla T Y (Abdalla et al., 2017) proposed a fuzzy control algorithm to improve the APF method, and the proposed problems were successfully solved. A fuzzy logic controller was used to control the movement of the robot, and a particle swarm optimization algorithm was used to optimize the membership function of the controller. Rostami S M H (Rostami et al., 2019) proposed an improved APF method to address the optimal path and solve the problems of local minima and unreachable targets in the APF algorithm, realizing effective robot obstacle avoidance without falling into local minima. Orozco-Rosas U (Orozco-Rosas et al., 2019) proposed a membrane evolution APF method for robot path planning, combining membrane calculation using a genetic algorithm and APF method to find suitable parameters, thus generating a feasible and safe path. This method consists of limited separated regions, in which there are several groups of parameters evolving according to biochemical inspiration to minimize the path length. Compared with classical APF methods, it shows better performance in path length. Jiachen Yang (Yang et al., 2022) proposes a Residual-like Soft Actor Critic (R-SAC) algorithm for agricultural scenarios, which improves the efficiency of reinforcement learning through offline experts experience pre-training methods, and optimizes the reward mechanism of the algorithm by using multi-step TD error, which solves the dilemma that may occur in the training process, and is a stable and efficient path planning method.

The author analyzes the above three problems in detail and proposes three improvement methods:

1. A target point distance factor is introduced into the attraction and repulsion potential field functions to reduce the resultant attraction and repulsion force received near a target point when the algorithm is far away;
2. An improved variable polynomial is used in the repulsion potential field function, which minimizes the distorted obstacle potential field when the robot is not near the target point and simultaneously ensures that the robot takes the global minimum at the target point;
3. The scope of the repulsion potential field is changed to an ellipse, and a fruit tree boundary potential field is added to reduce the environmentally potential field complexity so that the robot can avoid obstacles in advance without crossing the fruit tree boundary.

The effectiveness of the improved algorithm is verified through simulation and field tests.

## 2 Improved artificial potential field method with boundary constraints

### 2.1 Attractive potential field with distance factor introduced

The distance between the robot and a target point in a traditional APF method directly determines the attractive potential field function or the attractive force. When the distance between the robot and the target point is very large, the attractive potential field function or attraction will also become very large. In other words, the attraction plays a major role, while the repulsion plays a very small role in the robot motion control, which will easily lead to collisions between the robot and obstacles. To solve the collision risk of robots in an obstacle environment when considering the deviation of path planning, the attractive potential field function of the APF method is optimized, and a target point distance factor is added to reduce the attraction of the algorithm when the target point is far away. The improved attractive potential field function is defined as follows:


(1) $$\begin{array}{c} U_{att}\left(X\right)=\{\begin{array}{c} \frac{1}{2}k\cdot \rho^{2}\left(X,X_{g}\right),\rho \left(X,X_{g}\right)\leq d=2\rho_{0}\\ \frac{1}{2}k\cdot d\cdot \rho \left(X,X_{y}\right),\rho \left(X,X_{g}\right)>d=2\rho_{0} \end{array}\; \end{array}$$

where *k* is the attractive gain coefficient, *d* is a constant determined by the environment, *X*(*x*,*y*) is the current position of the robot, *ρ*(*X*,*X_g_*) is the distance between the robot and the target point, and *ρ*_0_ is the influence radius of the obstacle.

The improved attractive function is shown in Formula (2):


(2) $$\begin{array}{c} F_{att}\left(X\right)=\{\begin{array}{c} -k\cdot \rho \left(X,X_{g}\right)\cdot \nabla \left(X,X_{g}\right),\rho \left(X,X_{g}\right)\leq d=2\rho_{0}\\ -\frac{1}{2}k\cdot d\cdot \nabla \left(X,X_{g}\right),\rho \left(X,X_{g}\right)>d=2\rho_{0} \end{array} \end{array}$$

### 2.2 Improved elliptic repulsion potential field with variable polynomials

**Figure 1:**
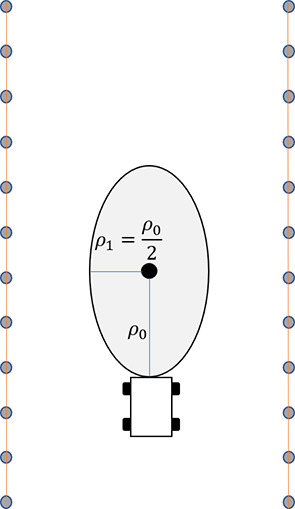
Influence range of the elliptic repulsion potential field.

In the actual operation process, a mowing robot is limited by the orchard environment and its own performance, so the obstacle repulsion potential field influence range is different from that of a traditional APF method. Therefore, the repulsion potential field influence range is improved as follows: the longitudinal distance of the influence range is increased so that the mowing robot can correct its direction in advance and enter the obstacle avoidance mode; the lateral distance of the influence range is reduced to ensure that the mowing robot can avoid obstacles safely. After modification, the influence range becomes oval, as shown in **Figure 1**:

In this study, the major axis and minor axis of the influence range of the repulsive potential field are *ρ*_0_ and $\rho_{1}=\frac{\rho_{0}}{2}$ .

By improving the repulsive potential field function, the local minimum and the oscillation around obstacles are solved. To address the problems in an APF, a method of adding a rotating force is adopted to improve the repulsion function (Gao et al., 2023) by applying a polynomial factor not less than zero to the repulsion potential field, which becomes zero when the robot reaches the target position. When the superimposed potential fields are all equal to zero at the target position, the robot position is the global minimum. This polynomial is the squares (Yang et al., 2016; Xin et al., 2022) of the distance from the robot to a target point. This form of the repulsive potential field greatly distorts the shape of the repulsive potential field when the robot is not near a target point while ensuring the global minimum of the target point. Therefore, in this study, an improved variable polynomial is used to minimize the distorted obstacle potential field when the robot is not near a target point and at the same time ensure that the robot has the global minimum at the target point. The improved repulsion potential field function is defined in Formula (3):


(3) $$\begin{array}{c} U_{rep}\left(x\right)=\{\begin{array}{c} \frac{1}{2}\eta \cdot \left[\frac{1}{\rho \left(X,X_{0}\right)}-\frac{1}{\rho_{0}}\right]\cdot \left[1-e^{\frac{\rho^{2}\left(X,X_{g}\right)}{R^{2}}}\right],X\epsilon \frac{{\left(x-x_{0}\right)}^{2}}{\rho_{0}^{\;2}}+\frac{{\left(y-y_{0}\right)}^{2}}{\rho_{1}^{\;2}}=1\\ 0,X\notin \frac{{\left(x-x_{0}\right)}^{2}}{\rho_{0}^{\;2}}+\frac{{\left(y-y_{0}\right)}^{2}}{\rho_{1}^{\;2}}=1 \end{array} \end{array}$$

where *η* is the repulsion gain coefficient, *ρ*_0_ is the major axis of the influence range of the obstacle, $\rho_{1}=\frac{\rho_{0}}{2}$ is the minor axis of the influence range of the obstacle, *R* is the radius of the robot, *X*_0_(*x*_0_,*y*_0_) is the position of an obstacle, *X_g_* (*x_g_*,*y_g_*) is the position of a target point, *ρ*(*X*,*X*_0_) is the Euclidean distance between the current position of the robot and the position of obstacle *X*_0_ and *ρ*(*X*,*X*_0_) is the Euclidean distance between the robot and target point. When the robot moves to the target position, the total potential field *U_total_* (*X*) is equal to zero. Therefore, when the robot moves to the target position, the robot will stop moving at the target position when the speed drops to zero, so the total potential field of the robot at the target position is equal to zero.

The improved repulsion function is shown in Formula (4):


(4) $$\begin{array}{c} F_{rep}\left(X\right)=\{\begin{array}{c} F_{rep1}\left(X\right)+F_{rep2}\left(X\right),X\epsilon \frac{{\left(x-x_{0}\right)}^{2}}{\rho_{0}^{\;2}}+\frac{{\left(y-y_{0}\right)}^{2}}{\rho_{1}^{\;2}}=1\\ 0,X\notin \frac{{\left(x-x_{0}\right)}^{2}}{\rho_{0}^{\;2}}+\frac{{\left(y-y_{0}\right)}^{2}}{\rho_{1}^{\;2}}=1 \end{array} \end{array}$$

where Formula (5) *F_rep_*_1_(*X*) means that the robot is far away from an obstacle along the line connecting it with the obstacle, and it decreases with the decrease in the distance between the robot and the target point; Formula (6) *F_rep_*_2_(*X*) means that the robot approaches the target position along the line connecting the robot and target position.


(5) $$\begin{array}{c} F_{rep1}\left(X\right)=\frac{1}{2}\eta \cdot \frac{1}{\rho^{2}\left(X,X_{0}\right)}\cdot \left[1-e^{\frac{\rho^{2}\left(X,X_{g}\right)}{R^{2}}}\right]\cdot \nabla \left(X,X_{0}\right),X\epsilon \frac{{\left(x-x_{0}\right)}^{2}}{\rho_{0}^{\;2}}+\frac{{\left(y-y_{0}\right)}^{2}}{\rho_{1}^{\;2}}=1 \end{array}$$

(6) $$\begin{array}{c} F_{rep2}\left(X\right)=\frac{1}{2}\eta \cdot \left[\frac{1}{\rho \left(X,X_{0}\right)}-\frac{1}{\rho_{0}}\right]\cdot \left[e^{\frac{\rho^{2}\left(X,X_{g}\right)}{R^{2}}}\cdot \frac{2\rho \left(X,X_{g}\right)}{R^{2}}\right]\cdot \nabla \left(X,X_{g}\right)\\ ,X\epsilon \frac{{\left(x-x_{0}\right)}^{2}}{\rho_{0}^{\;2}}+\frac{{\left(y-y_{0}\right)}^{2}}{\rho_{1}^{\;2}}=1 \end{array}$$

### 2.3 Introduction of a fruit tree boundary potential field

**Figure 2:**
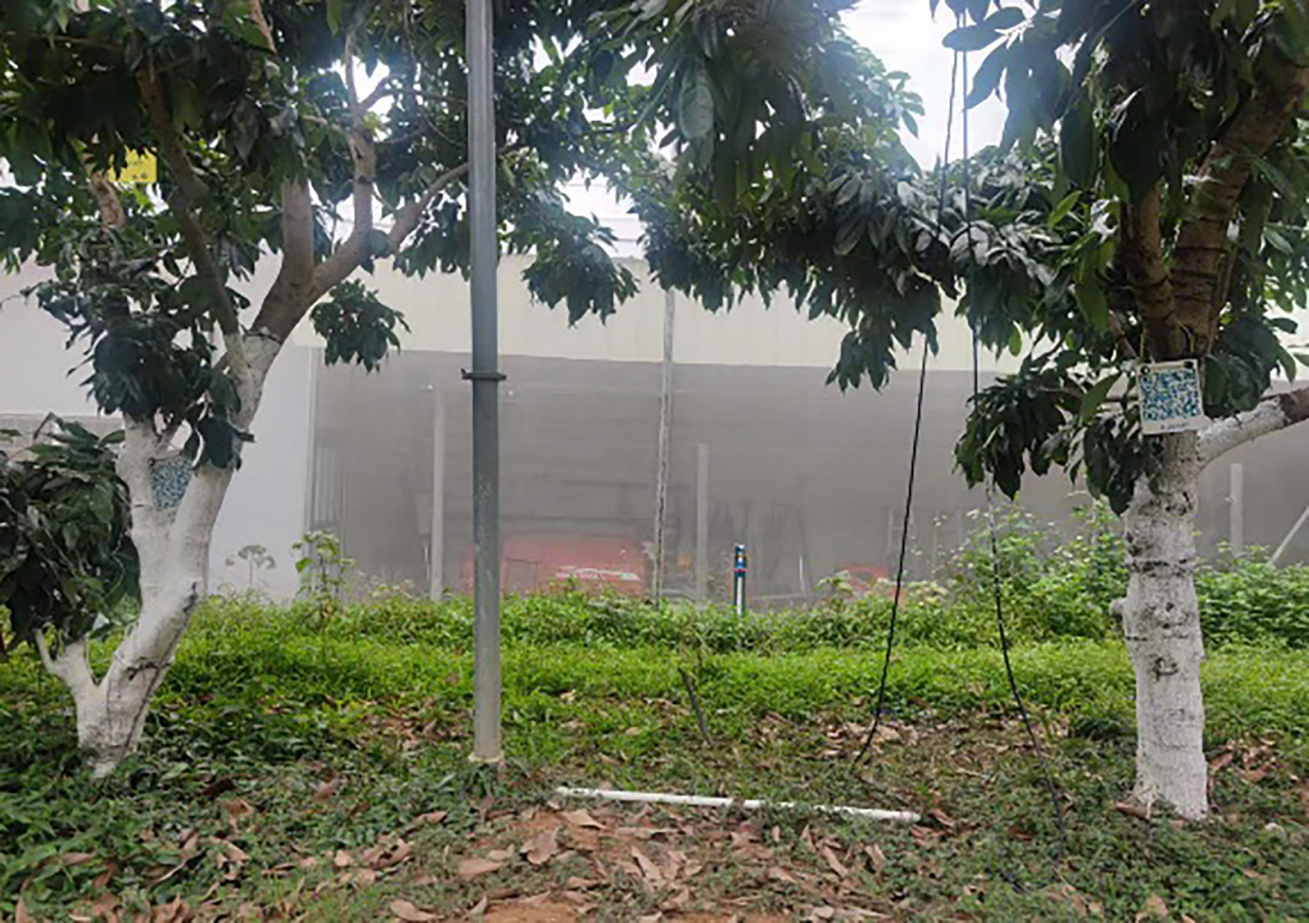
Facilities installed in fruit tree intervals.

**Figure 3:**
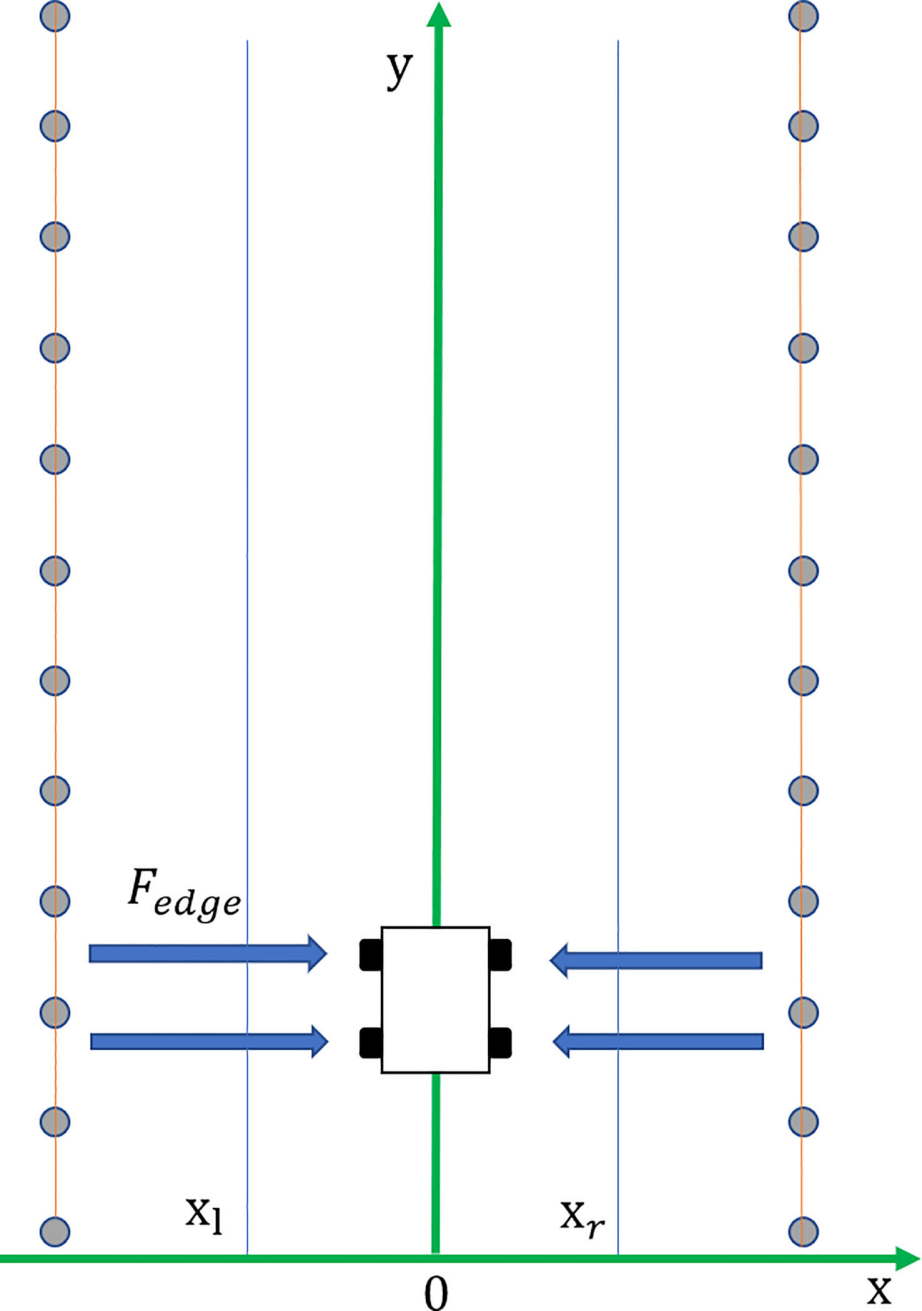
Boundary potential field.


When a mowing robot operates in an actual orchard, it needs to consider the influence of the surrounding environment while considering the obstacles. When the mowing robot moves to avoid obstacles, it cannot hit the fruit trees. In most orchards, facilities such as water and fertilizer irrigation and green prevention and control are installed among the fruit trees, as shown in **Figure 2**. If the fruit trees are regarded as individual obstacles, a large number of obstacles will easily make the mowing robot fall into the local minimum, and it is impossible to drive to the target point. At the same time, according to the operating characteristics of the mowing robot, it is easy to damage the facilities when driving into a fruit tree row. Therefore, adding a repulsive potential field to each fruit tree row as a boundary can effectively reduce the environmental potential field complexity and prevent orchard facilities from being damaged. According to mowing robot operating experience, the fruit tree boundary is the area with the highest risk factor, followed by the middle area of the path, as shown in **Figure 3**. According to the above distribution of the path danger degree, a path boundary potential field function is considered in sections. When the mowing robot is located in the area between paths, a function with a relatively gentle change trend is adopted; however, when it is close to the fruit tree boundary area, because of the high risk coefficient, a function with a large change trend is adopted. Based on the above factors, the orchard path is divided into four parts, and a fruit tree boundary potential field function is established as shown in Formula (7):

(7) $$\begin{array}{c} U_{edge}\left(X\right)=\{\begin{array}{c} \begin{array}{c} \eta_{edge}\cdot v\cdot e^{x-x_{l}},x\leq x_{l}\\ \frac{1}{3}\eta_{edge}\cdot x^{2},x_{l}<x<0\\ -\frac{1}{3}\eta_{edge}\cdot x^{2},0\leq x<x_{r} \end{array}\\ \eta_{edge}\cdot v\cdot e^{x-x_{r}},x_{r}\leq x \end{array} \end{array}$$

where *η_edge_* is the potential energy gain coefficient near the fruit tree boundary, $\frac{1}{3}\eta_{edge}$ is the potential energy gain coefficient of the middle part of the path, $X_{l}=-\frac{L}{4}$ is the dividing line near the left boundary, $X_{r}=\frac{L}{4}$ is the dividing line near the right boundary, and *L* is the path width.

In summary, the total potential field function of the improved APF method is:


(8) $$\begin{array}{c} U_{total}\left(X\right)=U_{att}\left(X\right)+\sum_{i=1}^{n}U_{rep}\left(X\right)+U_{edge}\left(X\right) \end{array}$$

## 3 Algorithm test and result analysis

To verify the effectiveness of the improved APF method designed in this study. Written the improved algorithm, and validated the code through the MATLAB simulation platform, defining a path planning evaluation model. The simulation results of the improved algorithm are compared with those of the traditional artificial potential field method, and the practical test of the improved artificial potential field method is conducted on the self-developed mowing robot platform.

### 3.1 Path planning evaluation model

To directly evaluate the quality of different path planning methods, a path planning evaluation model is established in combination with a practical application, which mainly includes the following three key evaluation parameters:

#### 3.11 Path planning evaluation

The primary goal and basic requirement of path planning is to generate a safe collision-free path. If the robot collides with an obstacle or cannot reach the target during the path planning, the path planning is invalid, which is a typical 0-1 problem.


(9) $$\begin{array}{c} f_{success}=\{\begin{array}{c} 1,success\\ 0,fail \end{array} \end{array}$$

#### 3.12 The total length of the planned path

When the robot actually moves and runs, the total length of the planned path can be equated to the cost of energy and time consumed by the robot. The shorter the total length of the planned path is, the better the path planning result. Assuming that the planned path is divided into *N* sections and the path length of time period *i* is *S_i_*, the total length of the planned path is:


(10) $$\begin{array}{c} S_{total}=\sum_{i=1}^{N}S_{i} \end{array}$$

#### 3.13 Maximum turning angular velocity

In the real working environment space, the path planned by the robot is often a curve due to the existence of obstacles, so it is easy to know that the course of the robot changes in real time. The heading angular velocity of the robot is the first derivative of the heading angle with respect to time. The smaller the angular velocity of the robot is, the smoother the planned path, and the better the stability and maneuverability of the robot.

Let the heading of the *i* time period be *θ_i_* and the heading of the *i* + 1 time period be *θ_i_*_+1_; then, the heading angular velocity of the robot is:


(11) $$\begin{array}{c} \Delta \theta_{i}=\frac{\theta_{i+1}-\theta_{i}}{step_{i}},\left(i=1,2,\ldots N-1\right) \end{array}$$

where *step_i_* is the time consumed in planning period *i*. In the whole path planning cycle, the maximum absolute value of the heading difference may be taken as the maximum turning angular velocity, that is,


(12) $$\omega_{\max}=\max\{\Delta \theta_{1},\ldots ,\Delta \theta_{i},\ldots ,\Delta \theta_{n-1}\},\;\left(i=1,2,\ldots ,N-1\right)$$

Based on the above three parameters, the path planning is evaluation model determined, and the evaluation function value is *VF*, as shown in Formula (13):


(13) $$\begin{array}{c} VF=f_{success}\cdot \left(\frac{r_{1}}{S_{total}}+\frac{r_{2}}{\omega_{\max}}\right) \end{array}$$

where *r*1 and *r*2 are greater than zero and satisfy *r*1 + *r*2 = 1. By definition, the larger *VF* is, the higher the quality of the planned path.

### 3.2 Test steps and parameter settings

#### 3.2.1 Simulation test steps and parameter settings

The lawn mower robot obstacle avoidance path planning of based on the improved APF method can be divided into the following steps:

S1, setting the positions of the starting point and the target point of the mowing robot, initializing the parameters, and establishing an environmental model around the robot using sensors mounted on the robot for environmental perception;
S2, calculating the attraction potential field function;
S3, calculating the repulsion potential field function;
S4, calculating the boundary potential field function;
S5, calculating the magnitude and direction of the attraction and repulsion exerted by the robot, calculating the components of the attraction and repulsion in the horizontal direction and the vertical direction, and determining the magnitude and direction of the total potential force exerted by the robot;
S6, setting the mowing robot moving step and updating the robot coordinates.

(14) $$\begin{array}{c} x\left(k+1\right)=x\left(k\right)+l\cos\theta \end{array}$$

(15) $$\begin{array}{c} y\left(k+1\right)=y\left(k\right)+l\sin\theta \end{array}$$

Guided by the total potential force of the APF method, the robot moves to a target point and the coordinates are updated. When the robot does not reach the target point, it continues to run under the combined force. When the mowing robot reaches the target point, it stops running. Thus, the planning path that meets the mowing robot operating requirements is obtained.

The attraction gain coefficient *k* =15, the repulsion gain coefficient *η* = 20 and the boundary repulsion gain coefficient *η_edge_* = 35 in the improved APF method are set through continuous experimental tests, and the major axis *ρ*_0 =_ 2 _m_, minor axis $\rho_{1}=\frac{1}{2}\rho_{0}=1\text{m}$ , and step length *l* = 0.05 m. The traditional APF method has an attractive gain coefficient *k* = 15, a repulsive gain coefficient *η* = 20, an obstacle influence range *ρ*_0 =_ 2 m, and a step size *l* = 0.05 m. Considering the distance between the robot and the target, the repulsion potential field function is improved as a polynomial factor with an index *m* =1, and the other parameters are the same.

#### 3.2.2 Real machine test steps and parameter settings

**Figure 4:**
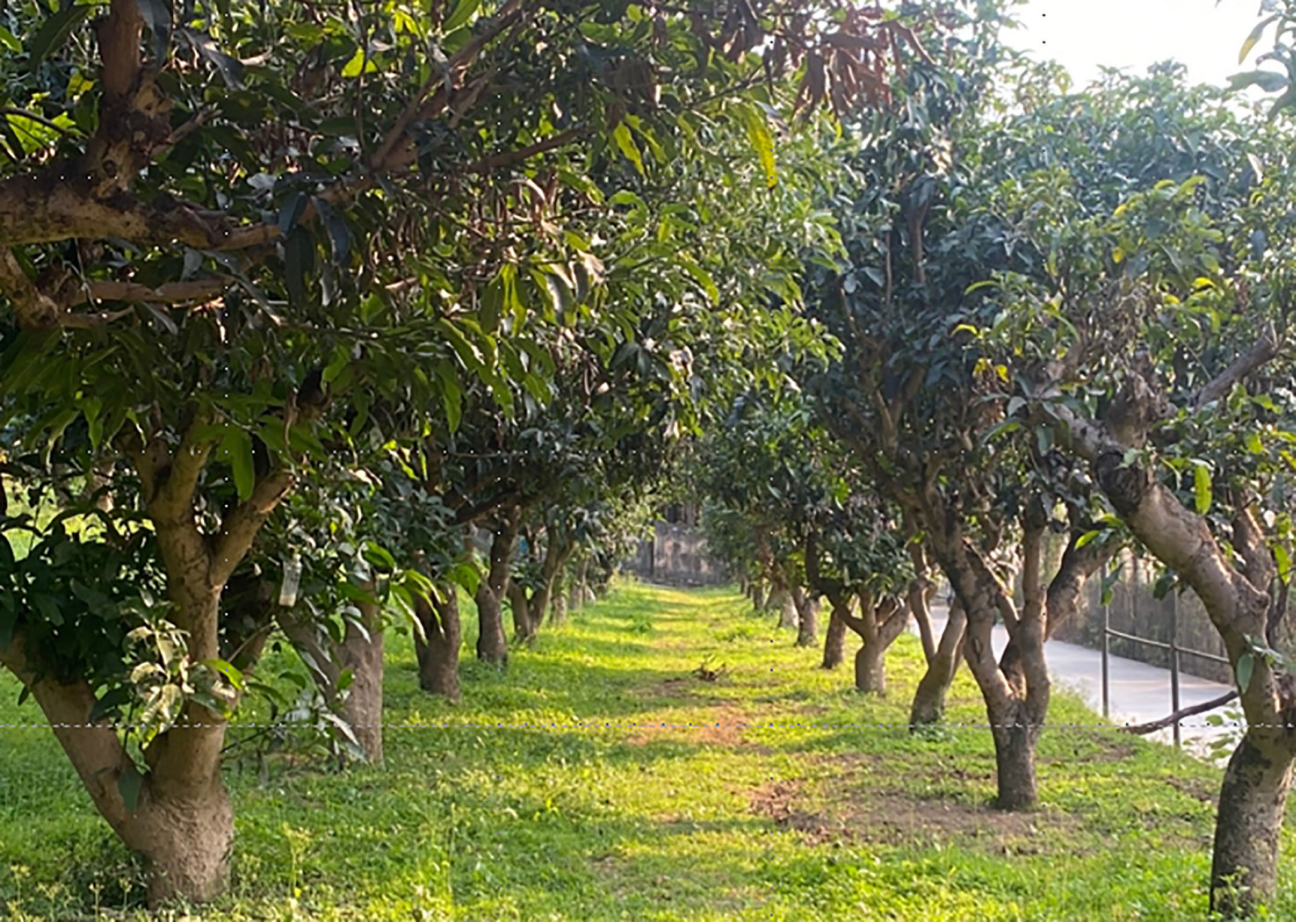
Orchard environment.

**Figure 5:**
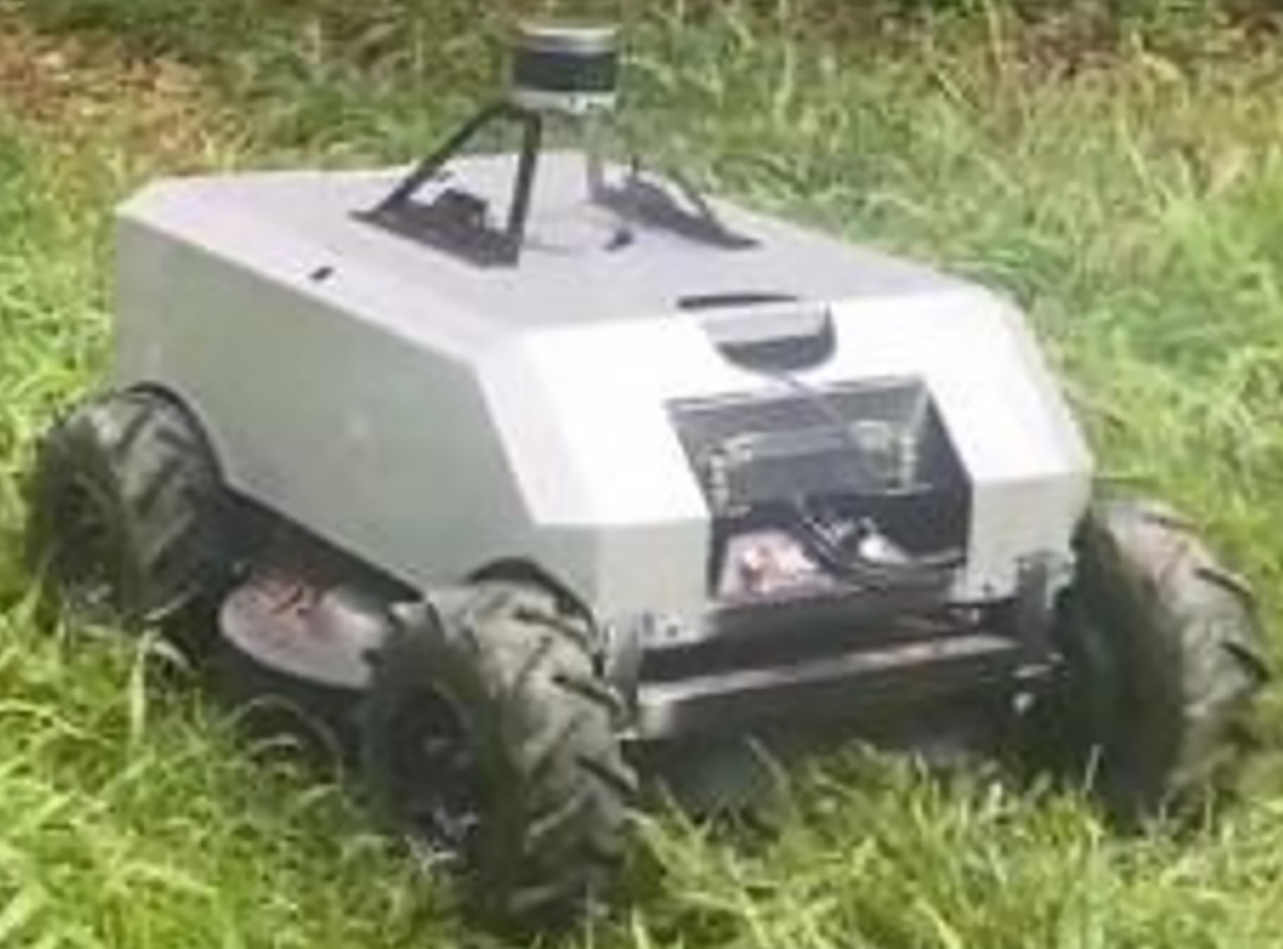
Real machine platform.

Using a self-developed mowing robot platform, a real machine verification test of the improved APF method is carried out, and a four-wheel electric differential structure is used. Equipped with 16-wire mechanical LIDAR, it can perceive the 360° environment around the lawn mower. The mowing robot use GPS to obtain global absolute position information and fuse IMU high-frequency body posture information to realize the navigation and positioning. The mowing robot measures the wheel speed through the rotary encoder to receive real-time feedback and control the vehicle speed, and obtain the actual trajectory value through the path tracking algorithm. The experimental environment is a modern standard orchard in the school, shown in **Figure 4**, with a spacing of 4 m and a length of 25 m. The real machine platform is shown in **Figure 5**.

According to the research objectives and content, the real-time obstacle avoidance experiment steps of the mowing robot are as follows:

1. A starting position (0, 0) and a target position (0,20) for the mowing robot are set according to an actual application scene;
2. To verify the applicability of the mowing robot, the scene is set according to the simulation test, obstacles are randomly placed between the starting position and the target position, and the obstacle position information is collected and recorded.
3. The mowing robot and all its instruments and equipment are started at the initial position, and the path planning algorithm based on the improved APF method is run to make the robot move autonomously in the obstacle scene set in step (2) and realize real-time obstacle avoidance;
4. A data acquisition program is run during the experiment and the GPS and IMU are used to collect the experimental data of the mowing robot during autonomous operation;
5. The real vehicle experimental data collected in step (4) are analyzed and compared with the planned path to verify the feasibility and effectiveness of the designed improved APF method.

In the Visual Studio Code software environment, the improved APF method is compiled into a Python program, uploaded to the vehicle controller, and the program is run in the set obstacle environment for a real vehicle test. The algorithm parameters are set as follows: attractive gain coefficient *k* = 15, repulsive gain coefficient *η* =30, boundary repulsive gain coefficient *η_edge_* = 40, obstacle influence range major axis *ρ*_0 =_ 3 _m_, minor axis $\rho_{1}=\frac{1}{2}\rho_{0}=1.5\text{ m}$ , and step length *l* = 0.1 m.

### 3.3 Simulation test results and analysis

**Scenario 1:**

In general, the obstacle environment is set as follows: there are *n* obstacles, with *n*=6, and the obstacle positions are *X*_0_=[3 0.2; 7 -0.4; 10 0.3; 13 -0.2; 15 0.5; 17 -0.4], the starting position of the robot is *X_s_* =[0 0.1], and the target position is *X_g_* =[20 0.1].

According to the established path planning evaluation model, the path quality planned using the different model algorithms under different scenarios is evaluated. The evaluation data are shown in **Table 1**:

**Table 1:** Scenario 1 path planning data quality evaluation under the same environment.

Case	*fcollision*	*S_total_* (*m*)	*ω_max_* (°/*s*)	*VF*
**Case 1**	1	21.72	72.8	0.0428
**Case 2**	1	20.34	15.9	0.0505

**Figure 6:**
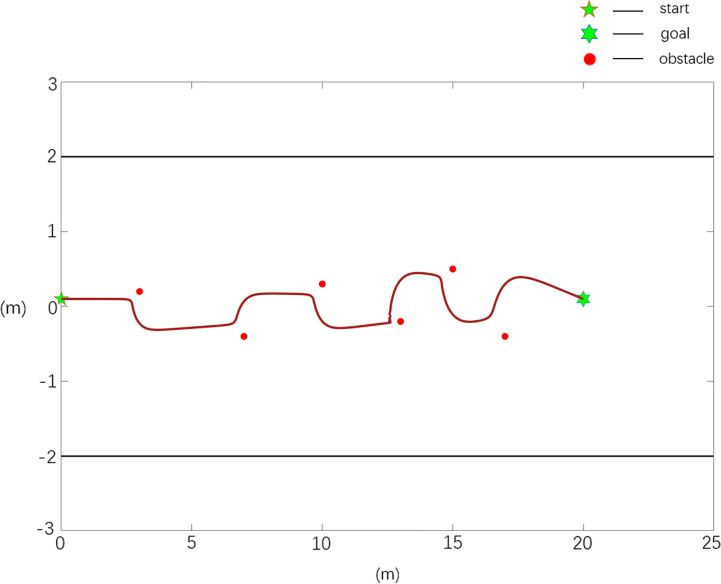
Scenario 1, case 1 test result using the traditional APF method.

**Figure 7:**
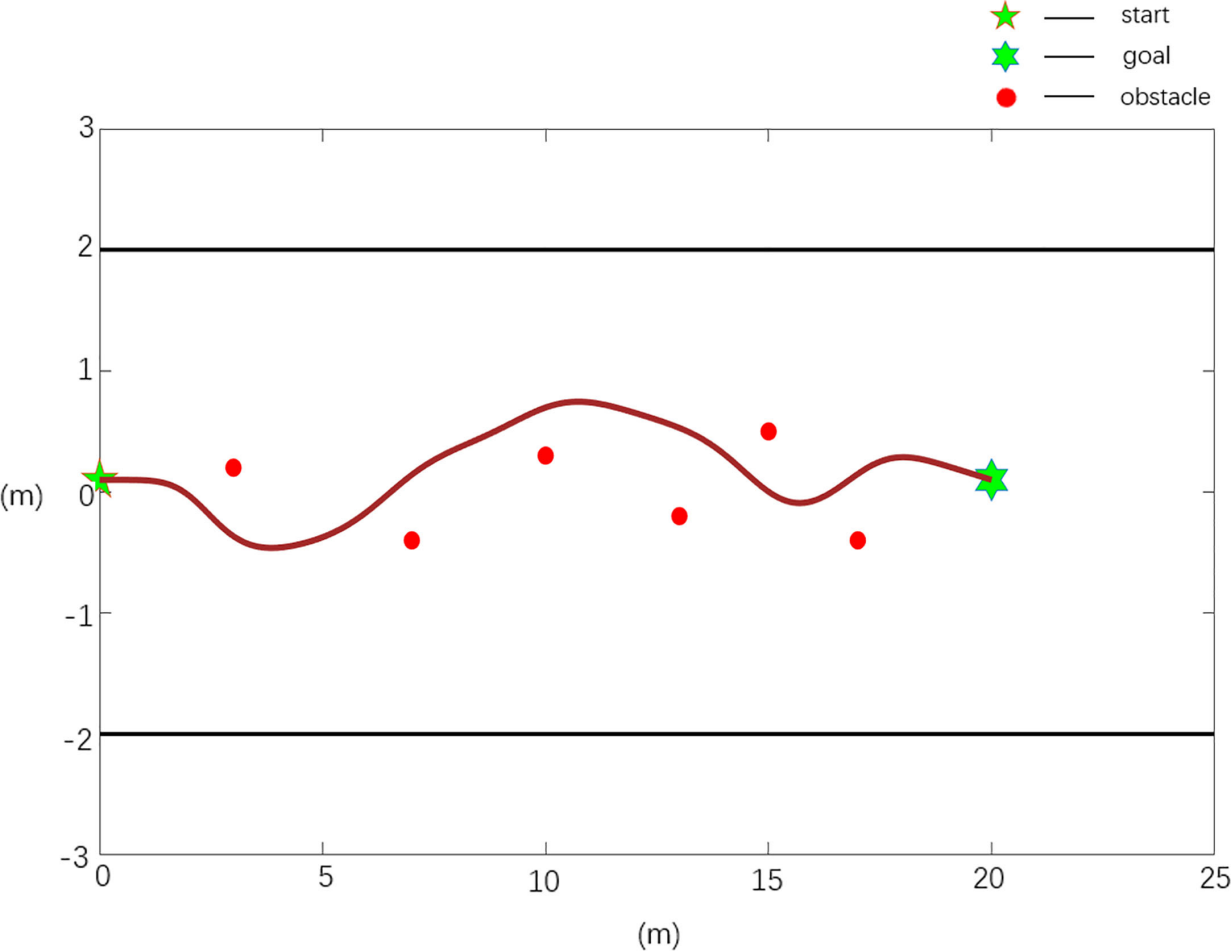
Scenario 1, case 2 test result 1 using the improved APF method.

The scenario 1 simulation results are shown in **Figures 6**, **7**, and the experimental results show that both the improved APF method and the APF method can realize collision-free effective path planning. Among them, there is slight oscillation in the planned path in **Figure 6**. There is no oscillation or jitter in the planned path shown in **Figure 7**. From **Table 1**, by comparing the parameters *S_total_* and *ω_max_*, it is found that the path length planned in Case 2 is the shortest, *ω_max_* is greatly reduced, and the evaluation function *VF* value is the largest, so the path planned in Case 2 is shorter, smoother and better in quality than that planned using the traditional APF method.

**Scenario 2:**

In the setting of an obstacle environment with a local minimum, there are *n* obstacles, *n*=6, and the obstacle positions are *X*_0_=[3 0.2; 7 -0.4; 13 -0.2; 15 0.5; 17 -0.4; 17 0.5], the starting position of the robot is *X_s_* =[0 0.1], and the target position is *X_g_* =[20 0.1].

According to the established path planning evaluation model, the path quality planned using the different model algorithms under scenario 2 is evaluated, and the evaluation data are shown in **Table 2**.

**Table 2:** Scenario 2 path planning data quality evaluation under the same environment.

Case	*fcollision*	*S_total_* (*m*)	*ω_max_* (°/*s*)	*VF*
**Case 1**	0	16.92	68.4	0
**Case 2**	1	20.24	10.1	0.0543

**Figure 8:**
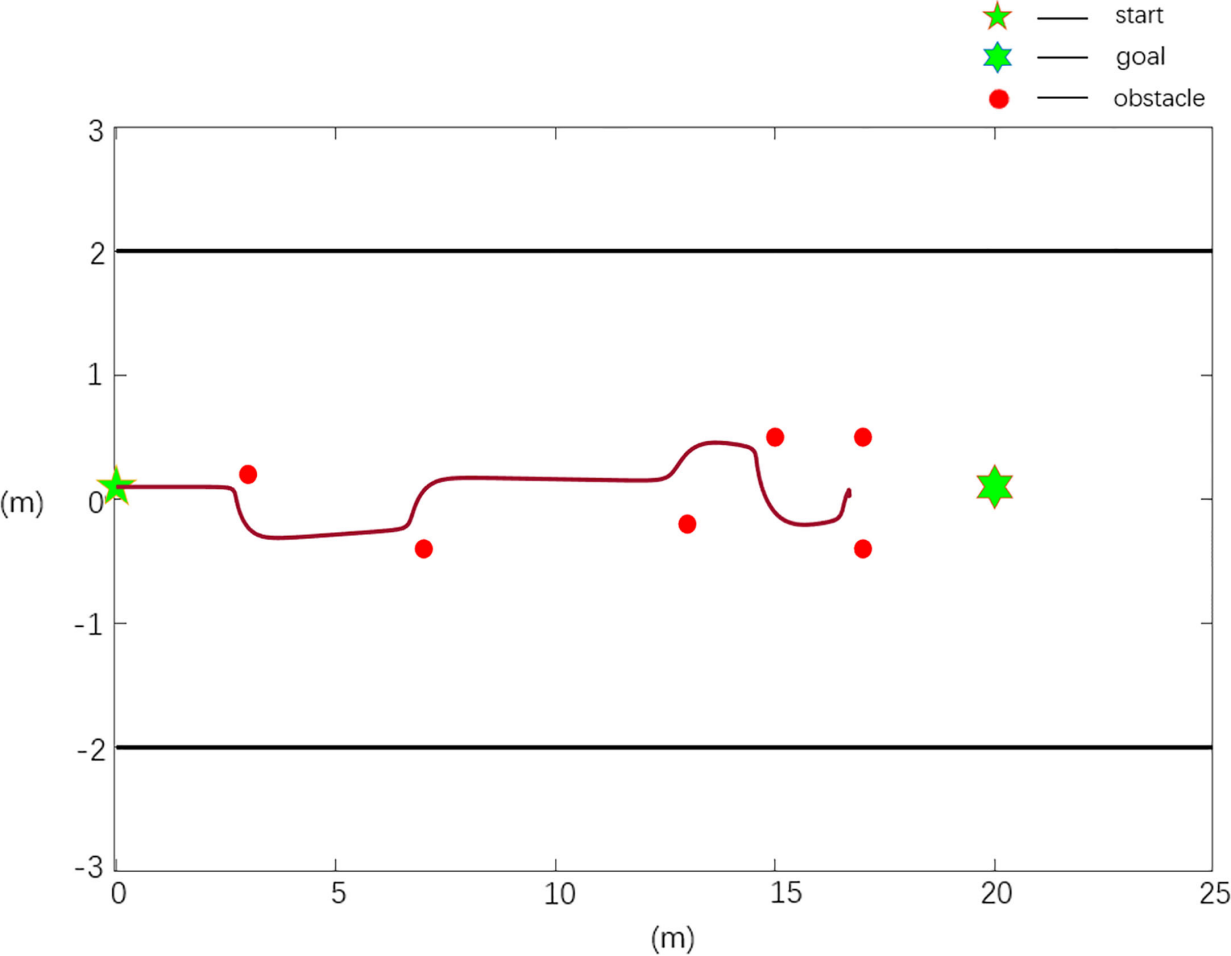
Scenario 2, case 1 test result using the traditional APF method.

**Figure 9:**
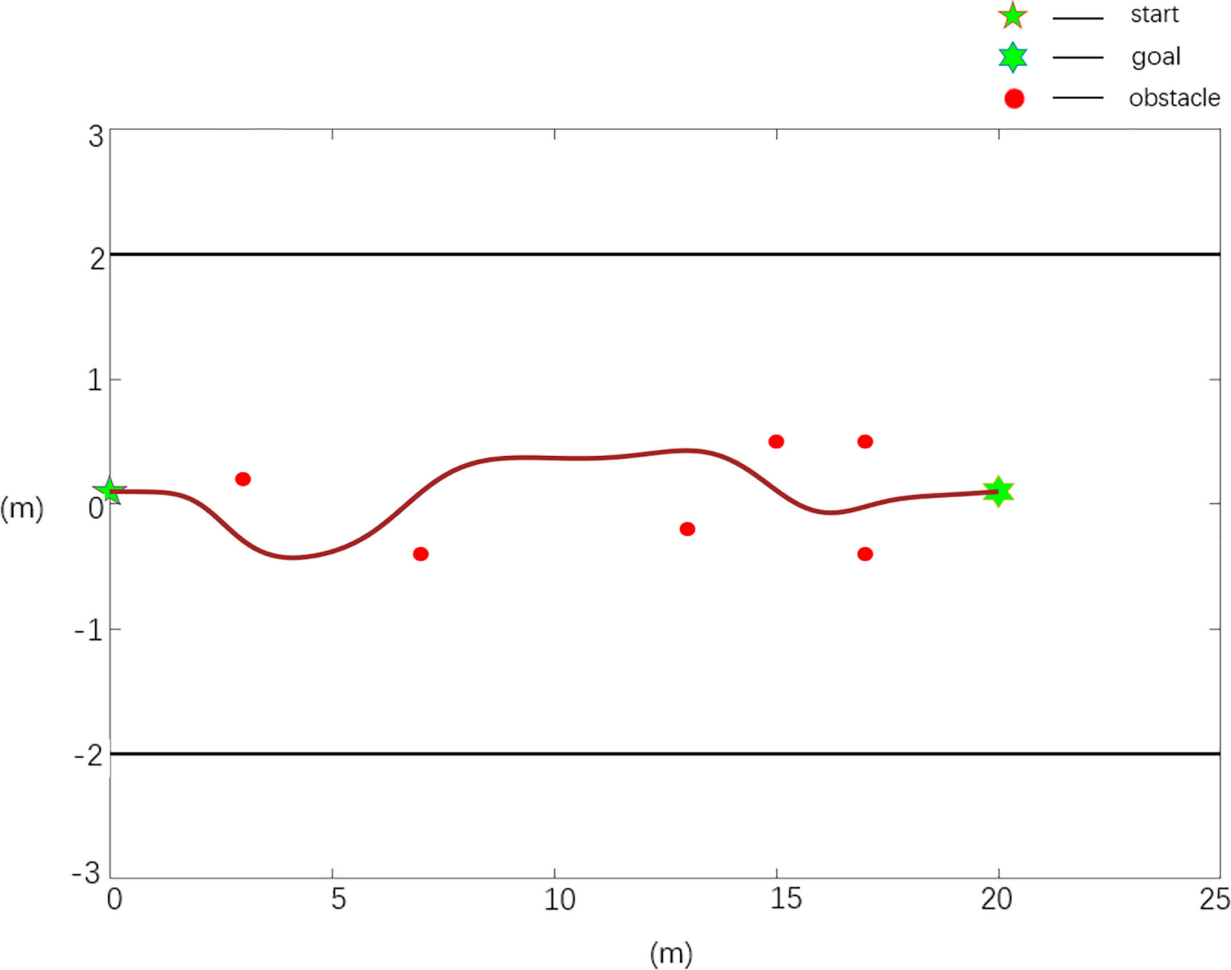
Scenario 2, case 2 test result using the improved APF method.

The scenario 2 simulation results are shown in **Figures 8**, **9**, and the experimental results show that the traditional APF method is ineffective in path planning. Among them, there is a local minimum problem in the planned path in **Figure 8**, and the robot cannot continue to move to the target position when it falls into a local minimum. It can be seen from **Figure 9** that the Case 2 method can realize effective path planning without collision, and in **Table 2**, compared with the parameter *ω_max_*, the path planned in Case 2 is smoother and has better quality.

**Scenario 3:**

In the obstacle environment in the case of boundary collision, there are *n* obstacles, *n*=6, and the obstacle positions are *X*_0_=[3 0.2; 5 -0.2; 7 -0.6; 9,-0.9; 10.5 -1.2; 12 -1.5], the starting position of the robot is *X_s_* =[0 0], and the target position is *X_g_* =[20 0].

According to the established path planning evaluation model, the path quality planned using the different model algorithms under different scenarios is evaluated, and the evaluation data are shown in **Table 3**.

**Table 3:** Scenario 3 path planning data quality evaluation under the same environment.

Case	*fcollision*	*S_total_* (*m*)	*ω_max_* (°/*s*)	*VF*
**Case 1**	0	13.69	51.95	0
**Case 2**	1	20.17	6.97	0.0589

**Figure 10:**
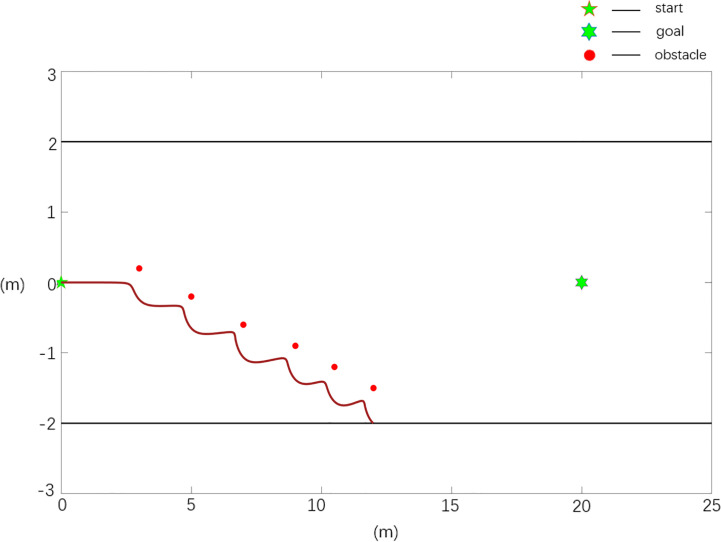
Scenario 3, case 1 test result using the traditional APF method.

**Figure 11:**
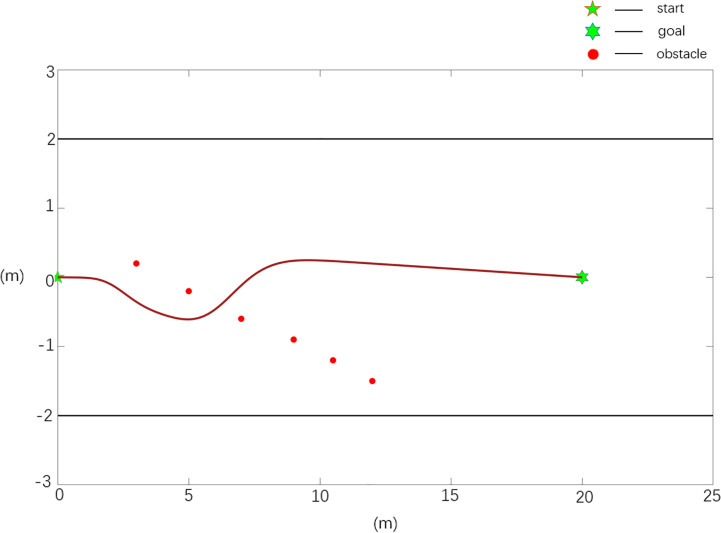
Scenario 3, case 2 test result using the improved APF method.

The scenario 3 simulation results are shown in **Figures 10**, **11**, and the experimental results show that the traditional APF method is ineffective in path planning. Among them, the path planned in **Figure 10** has a boundary collision problem, and the robot collides with the fruit tree boundary during obstacle avoidance, resulting in obstacle avoidance failure. As seen from **Figure 11**, the Case 2 method can realize effective path planning without collision and will not collide with the fruit tree boundary. Based on the experimental results and analysis of scenario 3, the designed Case 2 method can not only effectively realize collision-free path planning, overcome the oscillation or jitter phenomenon in the path planning process, and effectively solve the problem that the robot easily falls into a local minimum but also avoid the boundary collision problem in the obstacle avoidance process and has the best comprehensive performance.

### 3.4 Real machine test results and analysis

**Scenario 1:**

**Figure 12:**
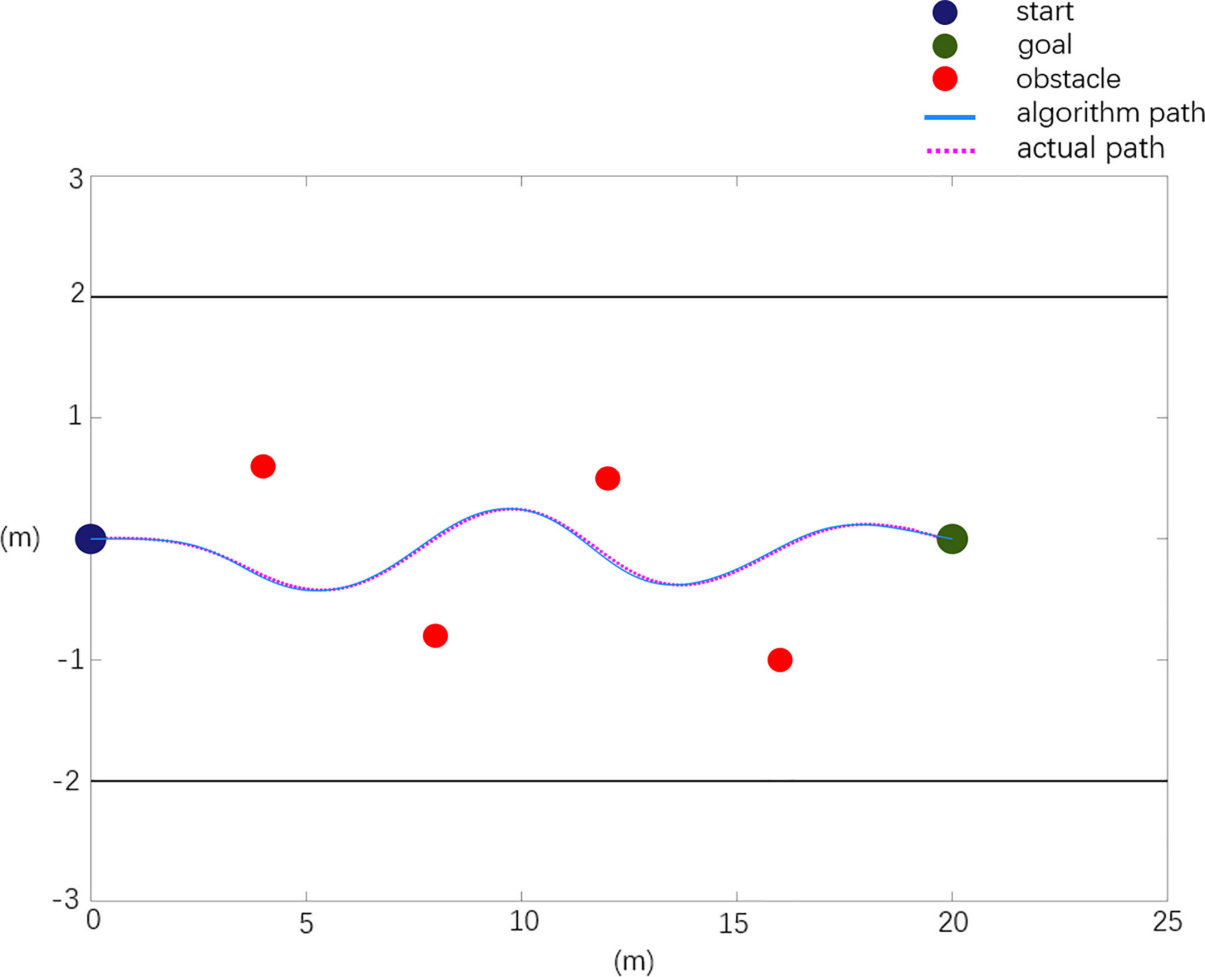
Real machine obstacle avoidance verification scenario 1.

In general, in the obstacle environment, the actual layout of obstacle position information is *X*_0_=[4.0 0.6; 8.0 -0.8; 12.0 0.5; 16 -1.0]. The experimental results are shown in **Figure 12**. Among them, the dark blue point in the figure is the starting point of the mowing robot, the green point is the target point, the red points are obstacles, the black straight line is the orchard boundary, the blue curve represents the reference path planned based on the improved APF method, and the purple dotted line represents the experimental results of obstacle avoidance for the real vehicle.

The experimental results of scenario 1 obstacle avoidance verification are analyzed, and the analysis data are shown in **Table 4**.

**Table 4:** Real machine obstacle avoidance verification experimental data analysis.

Path	Planning path	Actual path
Path length (m)	20.20	20.725
Maximum rotation angle (degree)	5.705	6.972
Maximum relative deviation in x direction (m)	0	0.137
Maximum relative deviation in y direction (m)	0	0.051

**Scenario 2:**

**Figure 13:**
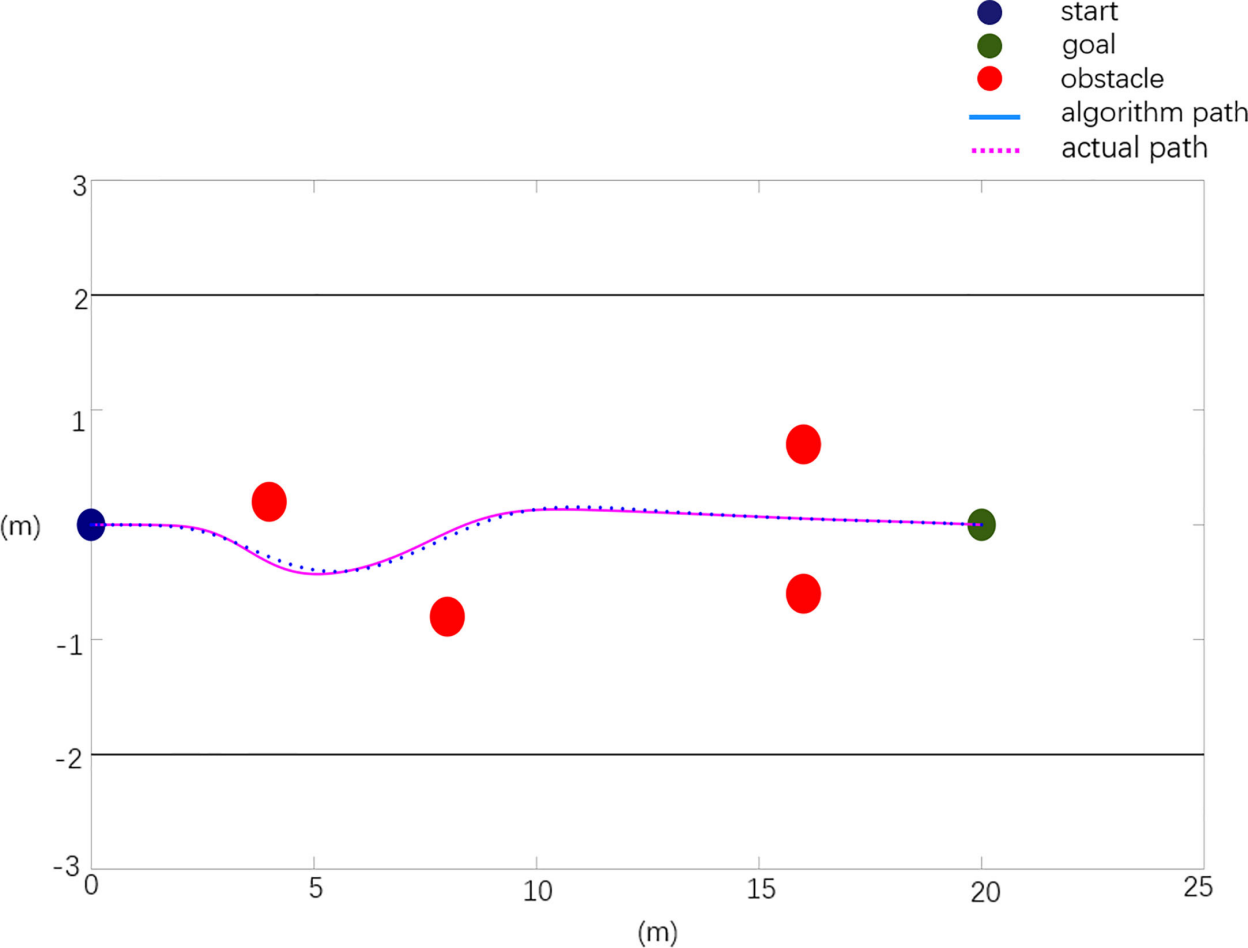
Real machine obstacle avoidance scenario 2 verification.

When there is a local minimum, the actual obstacle position information is *X*_0_=[4.0 0.2 8.0 -0.8; 16.0 0.7; 16 -0.6]. The experimental results are shown in **Figure 13**.

The experimental results of obstacle avoidance verification in scenario 2 are analyzed, and the analysis data are shown in **Table 5**.

**Table 5:** Real machine obstacle avoidance verification experimental data analysis.

Path	Planning path	Actual path
Path length (m)	20.12	20.592
Maximum rotation angle (degree)	5.63	4.975
Maximum relative deviation in x direction (m)	0	0.105
Maximum relative deviation in y direction (m)	0	0.048

**Scenario 3:**

**Figure 14:**
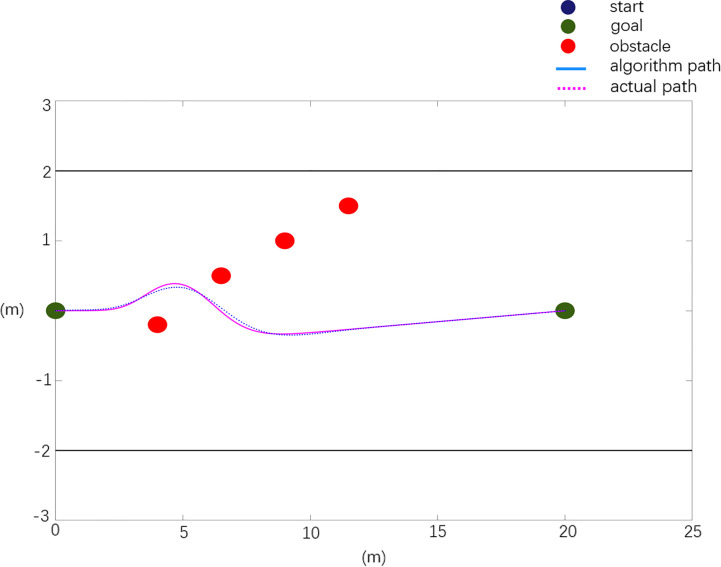
Real machine obstacle avoidance verification scenario 3.

In general, in the obstacle environment, the actual layout of obstacle position information is *X*_0_=[4.0 -0.2; 6.5 0.5; 9.0 1.0; 11.5 -0.6]. The experimental results are shown in **Figure 14**.

The experimental results of obstacle avoidance verification in scenario 3 are analyzed, and the analysis data are shown in **Table 6**.

**Table 6:** Real machine obstacle avoidance verification experimental data analysis.

Path	Planning path	Actual path
Path length (m)	20.17	20.730
Maximum rotation angle (degree)	9.23	8.572
Maximum relative deviation in x direction (m)	0	0.126
Maximum relative deviation in y direction (m)	0	0.053

The test results of scenario 1 are shown in **Figure 12** and **Table 4**. Compared with the planned path, the actual path has a length of 2.59% and a maximum rotation angle of 22.2%, with a maximum deviation of 0.137 m in the X direction and 0.051 m in the Y direction. As shown in **Figure 13** and **Table 5**, the test results of scenario 2 show that the actual path is 2.3% longer than the planned path, and the maximum rotation angle is 12% smaller, of which the maximum deviation in the X direction is 0.105 m and the maximum deviation in the Y direction is 0.048 m. The test results of scenario 3 are shown in **Figure 14** and **Table 6**. Compared with the planned path, the actual path length is 2.7% longer and the maximum rotation angle is 7.1% smaller, of which the maximum deviation in the X direction is 0.126 m and the maximum deviation in the Y direction is 0.053 m. The above situation shows that the gap between the actual path and the planned path is small, and the maximum displacement error is kept within 0.15 m, which meets the design needs.

The experimental results show that in an actual orchard environment, the mowing robot can effectively solve the local minimum problem and effectively avoid obstacles in the obstacle environment. The robot successfully completes the path planning from the initial position and avoids all obstacles to reach the target position safely. In the actual driving process, due to the influence of the orchard ground environment, the actual driving path deviates from the planned path, but it meets the control requirements of the mowing robot within the allowable control error.

## 4 Conclusion

The artificial potential field method has been widely used in local path planning because of its simple and real-time characteristics. To further improve the performance of the algorithm, many scholars have studied improving the method algorithms. In this study, the following methods are adopted: by improving the attractive field model, the problem of colliding with obstacles when the distance is too far and the attraction is too large is avoided; on the basis of the original repulsive force field, considering the influence of the relative position and speed between the target and the robot, a new repulsive function is introduced, and the repulsive potential field strength of obstacles near the target is reduced by adding a rotating force, thus solving the local minimum problem.

The actual operation requirements of a mowing robot require path planning in complex environments. This study combines the advantages of these two methods, considers the environmental constraints in an actual orchard, modifies the scope of the repulsive potential field, and introduces boundary potential field constraints to ensure that the algorithm can realize planning path that meets the actual operation requirements of mowing robots.

To address the shortcomings of traditional APF path planning algorithms, an improved APF path planning algorithm suitable for orchard mowing robots is proposed. The simulation experiment in this study can be divided into three parts: first, the robustness of the improved APF method compared with a traditional APF method is verified, and the planning path is smoother and shorter. Second, it is verified that the improved algorithm has a stronger ability to solve local minimum problems. Finally, an actual orchard working environment is simulated, and it is verified that the improved APF method has better adaptability to the orchard environment and can successfully avoid boundary collisions and complete obstacle avoidance to reach the target point. At the same time, according to the scenario set up in the simulation experiment, the corresponding practical verification experiment of the improved APF method is carried out. The experimental results verify the effectiveness and reliability of the improved algorithm. This provides a new method for the path planning of this kind of mowing robot working in orchard environments.

## Data availability statement

The raw data supporting the conclusions of this article will be made available by the authors, without undue reservation.

## Author contributions

All authors listed have made a substantial, direct, and intellectual contribution to the work and approved it for publication.
